# Infant buccal telomere length: associations with maternal distress in pregnancy and offspring temperament

**DOI:** 10.3389/fpubh.2025.1657714

**Published:** 2025-10-21

**Authors:** Anna E. Soerensen, Bea R. H. Van den Bergh, Marijke A. K. A. Braeken, Marion I. van den Heuvel, Dries S. Martens, Stijn Vos, Tim S. Nawrot

**Affiliations:** ^1^Centre for Environmental Sciences, Hasselt University, Hasselt, Belgium; ^2^Health Psychology Research Group, and Leuven Brain Institute, KU Leuven (University of Leuven), Leuven, Belgium; ^3^Rehabilitation Research Center, Faculty of Rehabilitation Sciences, Biomedical Research Institute, Hasselt University, Hasselt, Belgium; ^4^Tranzo Scientific Centre for Care and Wellbeing, Tilburg University, Tilburg, Netherlands; ^5^Department of Public Health and Primary Care, Occupational and Environmental Medicine, KU Leuven (University of Leuven), Leuven, Belgium

**Keywords:** telomere length, social, stress, infant, preschooler, behavior, temperament, work

## Abstract

**Introduction:**

Telomere length is considered a marker of biological aging, and is shown to be susceptible to exposures during gestational and early life, including maternal psychological distress, and to be connected with temperament. Here, we examine the influence of maternal psychosocial and work-related factors on infant telomere length, and how infant telomere length is associated with infant and preschooler temperament.

**Methods:**

147 mothers and their offspring from the Dutch Prenatal Early Life Stress (PELS) cohort participated in this study. Psychological distress and work-related factors were assessed with the State Trait Anxiety Inventory (STAI), Edinburgh Depression Scale (EDS), Effort-Reward Imbalance, Questionnaire on the Experience and Evaluation of Work (QEEW), and Copenhagen Psychosocial Questionnaire (COPSOQ) questionnaires at 16–23 gestational weeks. Offspring temperament was evaluated twice: first at median 4 (IQR: 3-5) months using the Infant’s Behavior Questionnaire (IBQ-R, very short form) and again at 4 years of age using the Children’s Behavior Questionnaire (CBQ-R, very short form). Multivariable adjusted linear regression models were used to test associations between maternal psychosocial and work-related factors in pregnancy, infant buccal telomere length at 3–5 months after birth and infant and preschooler temperament, adjusting for maternal age, BMI, and education, and offspring age and sex.

**Results:**

The PELS participants generally presented with low mental distress levels, with 9.5% of the participants having severe state anxiety scores and 5.4% of the participants having severe depression symptoms. Maternal STAI and EDS scores showed a positive correlation with preschooler negative affectivity. Maternal psychosocial and work-related factors exhibited no discernible associations with infant buccal telomere length (*p*’s ≥ 0.11). Additionally, infant buccal telomere length was not associated with offspring temperament. However, after adjusting for work-related stressors, social satisfaction showed a trend for significance on infant telomere length, with a 4.2% (95% CI: −0.31 to 8.76, *p* = 0.070) longer telomere length per 1 unit higher score in social satisfaction.

**Conclusion:**

Higher satisfaction with social life may have a positive impact on infant telomere length. Mild maternal stress during pregnancy does not seem to affect infant telomere length, nor is telomere length predictive of infancy and early childhood temperament.

## 1 Introduction

Telomere length shortens for every cell division and may be viewed as a biological marker of aging, being associated with disease susceptibility and life expectancy (1, 2). More recent research has also found telomere length to be connected to other areas, such as behavior (3–5). Telomere length is sensitive to oxidative stress, denoting that external exposures changing the levels of oxidative stress, such as air pollution (1, 6), green spaces (7, 8), and stress (9, 10) may influence telomere length. According to the Developmental Origins of Health and Disease (DOHaD) hypothesis, in-utero and early-life exposures can have long-lasting consequences for the health of the offspring (11–13). Indeed, studies have shown telomere length to be particularly susceptible to exposures during the early-life period (14–17). These exposures may already have an influence during pregnancy, as intrauterine growth restriction negatively influences placental telomere length, whereas preterm birth has been linked to longer telomeres at birth (18). Additionally, other conditions occurring during pregnancy, such as higher blood pressure (19), infection (20), and gestational diabetes mellitus (21, 22) show negative associations with newborn telomere length. Nevertheless, these effects are still under debate (20, 23). These inconsistencies may be attributable to stress-related mechanisms, as evidenced by findings that patients with preeclampsia exhibit elevated levels of depression, and those with hypertension during pregnancy display increased levels of depression, anxiety, and stress (24). Indeed, maternal distress during pregnancy has been particularly examined, with prenatal exposure to maternal psychological distress being associated with shorter telomere length in cord blood and leukocytes of the newborn (25–27). A meta-analysis found maternal psychological stress during pregnancy to be significantly associated with shorter cord blood telomere length in the newborn (28). However, to our knowledge, there are no studies examining prenatal exposure to maternal work-related factors on the offspring’s telomere length. This is despite several articles showing work-related factors, especially burnout, to have an influence on telomere length in adults (29–31).

Shorter leukocyte telomere length have also been found to be associated with lower scores of conscientiousness, neuroticism, reward dependence and harm avoidance (4), and higher scores of hostility (32) and pessimism (33) in adults. Seemingly only one study examined the link between telomere length and temperament in the early life, revealing that infant surgency lessened telomere length attrition, and regulation/effortful control was positively associated with telomere length (34). This is important, as childhood temperament is predictive of future life outcomes, such as cognitive performance (35) and behavioral problems such as internalizing and externalizing problems (36–40). Prenatal exposures to maternal stressors may therefore have long-lasting implications, affecting offspring telomere length, which, in turn, may be connected with offspring temperament.

The aim of our study was therefore to examine (1) how maternal psychosocial and work-related factors during pregnancy influence infant buccal telomere length at 3–5 months of age and (2) how infant buccal telomere length is related to offspring temperament.

## 2 Materials and methods

### 2.1 Study population

**Figure 1:**
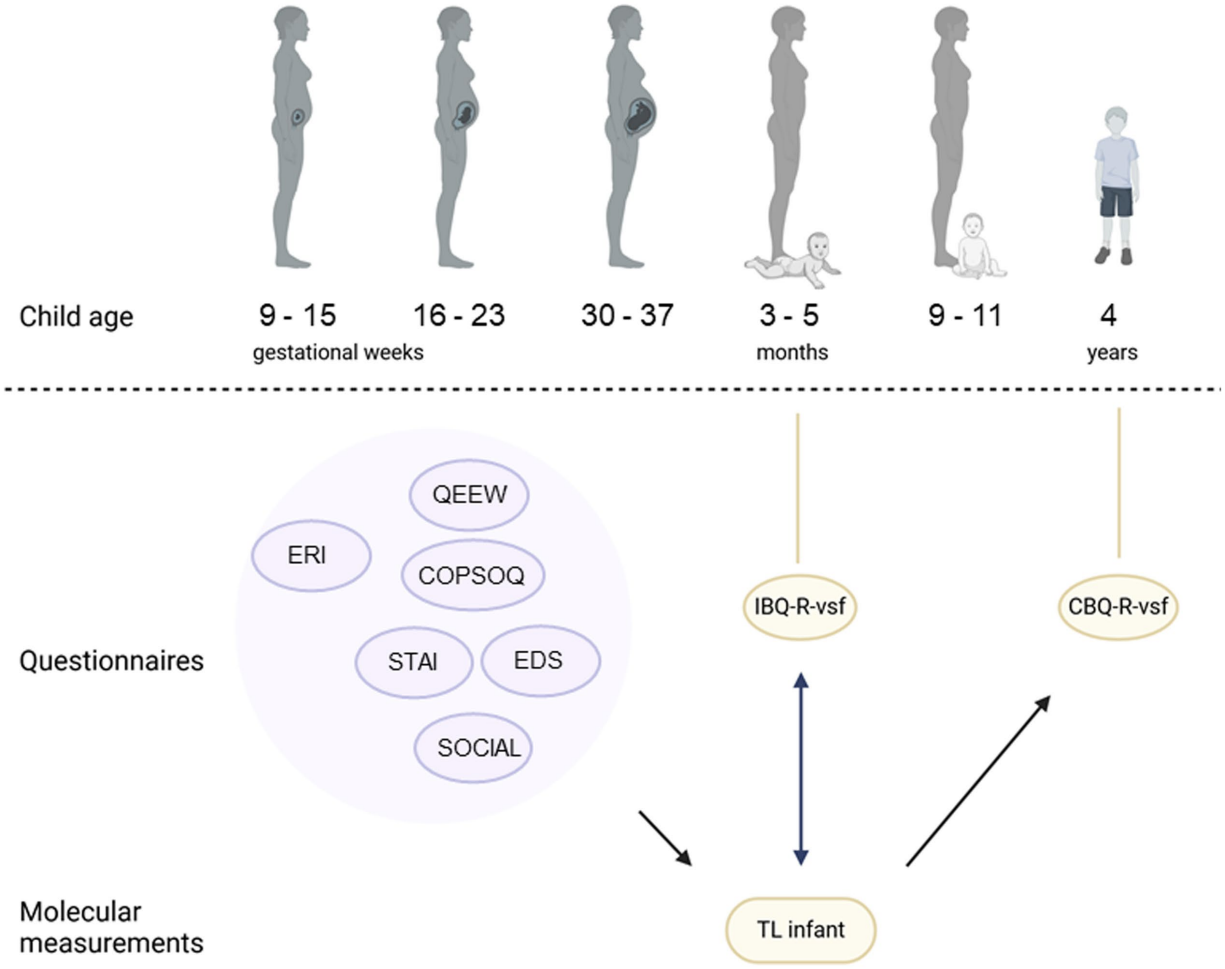
An overview of the PELS study cohort timepoints, alongside the used questionnaires, the telomere measurements and the associations examined. Round bubbles indicate questionnaires. Purple indicates maternal measurements, yellow indicates for the infant. STAI, Spielberger State Trait Anxiety Inventory; EDS, Edinburgh Postnatal Depression Scale; QEEW, Questionnaire on the Experience and Evaluation of Work; COPSOQ, Copenhagen Psychosocial Questionnaire; ERI: Effort-Reward Imbalance Questionnaire; IBQ-R-vsf, Infant Behavior Questionnaire Revised Very Short Form; CBQ-R-vsf, Child Behavior Questionnaire Revised Very Short form.

Participants from the Dutch Prenatal Early Life Stress (PELS) cohort were recruited in the Saint-Elisabeth hospital and four midwife practices in close vicinity to Tilburg (The Netherlands), where a total of 190 pregnant women were recruited during early- to mid-pregnancy from April 2009 to September 2010 (Figure 1). After an introduction of the study by the medical staff, interested participants left their contact information for the researchers. The researchers would then make an appointment with the participant for a first study visit. All participants gave informed written consent and the study has been approved by the Medical Ethical Committee of the Saint-Elisabeth hospital, Tilburg, The Netherlands (NL20261.008.07; EC.2012.31). All procedures were in accordance with the declaration of Helsinki. Participants reported information about birth date, work, education level and other demographics in a general questionnaire at the start of the study.

The study consisted of six consecutive measuring timepoints, at gestational age 9–15 weeks, 16–23 weeks, 30–37 weeks and postnatal 3–5 months, 9–11 months and 4 years (Figure 1). During the six sessions, different types of data were collected from the parents and, after birth, also from their infant and preschooler, this to relate prenatal exposure to maternal psychological distress with infant and preschooler motor, neurocognitive and behavioral outcomes.

Participants could choose whether they preferred paper and pencil questionnaire or an electronic version, using the Qualtrics software. Prepartum sessions mostly took place at the participants’ homes, unless the participant preferred meeting somewhere else, e.g., at work, at her parents’ home, or at Tilburg University Babylab. All postnatal measurements took place at the Tilburg University Babylab. Through midwives and hospital nurses, and consulting medical files, information on the delivery was also collected. This study mainly used measurements at gestational age 16–23 weeks, as this may be a more sensitive time period (1, 41), and when the offspring was 3–5 months and 4 years of age. Data on mother–offspring pairs was gathered 3–5 months after birth, where buccal swabs were taken from the infant, and temperament was assessed, as this age window captures early-emerging individual differences in regulatory and affective functioning (42, 43). At 4 years of age, the temperament of the preschooler was assessed via a maternal reported questionnaire (*n* = 114).

### 2.2 Psychosocial factors

#### 2.2.1 Maternal distress in pregnancy

Maternal self-reported state anxiety was measured using the Dutch-version of the Spielberger State – Trait Anxiety Inventory (STAI) (44, 45), containing 20 items for state anxiety, and 20 items for trait anxiety. All items are scored from 1 to 4 (1 = not at all, 4 = very much so) with a higher score indicating higher levels of anxiety. A cutoff score of ≥ 43 was used to define high anxiety (46, 47).

Maternal self-reported depressive symptoms were measured using the Edinburgh Postnatal Depression Scale (EDS), a questionnaire developed by Cox et al. (1987) (48) to screen mothers for depressive symptoms during pregnancy and the postnatal period. The questionnaire consists of 10 items scored from 0 to 3 about depressive symptoms of which a sum score can be calculated for overall depressive symptoms, with a higher score indicating higher levels of depression. A cutoff score of ≥ 12 was used (48, 49).

#### 2.2.2 Satisfaction with social life in pregnancy

Satisfaction with social life was measured by asking participants to what extent they were satisfied with their social contacts. They were asked separately for their satisfaction with respect to contact with close family members, contact with friends, and contact with people in their general environment. For each category, they indicated whether they were not very satisfied (score = 1), somewhat satisfied (score = 2) or very satisfied (score = 3). The sum scores for the three categories were calculated and used for further analysis as an indicator of general satisfaction with social life.

#### 2.2.3 Work-related factors in pregnancy

Participants filled out some subscales of the Copenhagen Psychosocial Questionnaire (COPSOQ) (50) as a measure of emotional stress and wellbeing in different work-related domains. We used the subscales ‘emotional demands’ and ‘possibilities for development’ (Cronbach’s *α*’s = 0.71–0.75). The ‘physical demands’ subscale of the Questionnaire on the Experience and Evaluation of Work (QEEW) (51, 52) was used for determining physical demands at work (Cronbach’s *α* = 0.91). Additionally, at 9–15 gestational weeks, the Effort-Reward Imbalance Questionnaire (ERI-Q) (53) was used to determine the effort/reward imbalance score for all participants (Supplementary eMethods 1). 138 out of 147 mothers were working during 9–15 gestational weeks (when the ERI was completed), and 139 during 16–23 weeks, and QEEW and COPSOQ analyses therefore had a *n* = 139, and ERI a *n* = 138.

#### 2.2.4 Sample collection and DNA extraction

At 3–5 months of age, infant buccal swabs were taken using the Cytosoft™ cytology brush (Thermo Fisher Scientific, Waltham, MA, United States), rotating it five times on one side and five times on the opposite interior cheek and placing the swab in proteinase K. DNA was extracted from buccal swabs using PrepIT-L2P kits (DNA Genotek Inc., Ottawa, ON, Canada) according to the manufacturer’s instructions in the Fritz Lipmann Institute (Jena, Germany) and stored at −20 °C before transfer to University Hospital Leuven, Belgium, where it was stored at −80 °C until the time of analysis at Hasselt University, Belgium. Yield and purity were checked using NanoDrop (Thermo Fisher; Supplementary eTable 1). Buccal swabs were chosen due to being non-invasive, and show correlations of 0.46 (54) to 0.57 (55) with the commonly-used leukocyte telomere lengths.

### 2.3 Infant buccal telomere length at 3–5 months

Infant buccal relative average telomere length was measured using a modified quantitative polymerase reaction (qPCR) method by Cawthon (56, 57), at Hasselt University, Belgium. This method is based on telomeres consisting of several thousand TTAAGGG repeats. By using a single copy gene, or a stable reference gene, one can measure the relative length of the telomere repeat copy number as compared to the single copy gene copy number, giving the relative telomere to single copy gene (T/S) ratio. This is more accurate than using the qPCR values for the telomere alone, as small differences in the template input will be adjusted for when compared to the stable gene. DNA was diluted to 2 ng/μL using QUBIT™ fluorometer (Invitrogen), using 5 ng of DNA per qPCR reaction. In a reaction volume of 10 μL, the final qPCR mixture concentrations for the telomere plates were 1X KAPA SYBR® FAST (low rox) mastermix, 2 μM dithiothreitol (DTT), 100 nM TelG (ACACTAAGGTTTGGGTTTGGGTTTGGGTTTGGGTTAGTGT) and 100 nM TelC (TGTTAGGTATCCCTATCCCTATCCCTATCCCTATCCCTAACA), which were manually added. The cycling conditions for the telomere plates were 1 cycle at 95 °C for 3 min, 2 cycles at 94 °C for 3 s and 49 °C for 15 s, 30 cycles at 94 °C for 3 s, 62 °C for 5 s and 74 °C for 10 s. The single-copy gene, human *β* globin (HBG), mixture contained 1X KAPA SYBR® FAST (low rox) mastermix, 450 nM HBG1 (GCTTCTGACACAACTGTGTTCACTAGC) and 450 nM HBG2 (CACCAACTTCATCCACGTTCACC) at the final concentration, and were measured with the following qPCR cycling program: 1 cycle at 95 °C for 3 min, 40 cycles at 95 °C for 3 s and 58 °C for 15 s. Both cycling programs ended with a melt curve. Samples were measured in triplicate using Quantstudio 5 (Applied Biosystems) in a 384-well format using fast mode. Two 6-point serial 1:3 dilutions consisting of pooled samples and one individual sample, and 5 interrun calibrators (IRCs) were included in triplicate. Amplification curves were visually inspected in QuantStudio™ Design and Analysis Software v1.5.2 and individual technical replicates were removed if the Ct variation was more than 0.3. Ct thresholds were set at 0.3. qBase plus (Biogazelle, Zwijnaarde, Belgium) was used for processing and normalization to the HBG gene and interrun variation, ending with the normalized relative quantities (CNRQs) of each sample. The calculations and further information can be found in Supplementary eMethods 2. The intraclass correlation coefficient (ICC) was used for measuring repeatability of both the triplicates and the plates, using the *rptR* R package (58), with a 95% confidence interval (CI). The ICC was measured for the telomere CT measurements, the single-copy CT measurements and the relative telomere length measurements of the samples. These were, respectively, 0.991 (0.989 to 0.992), 0.975 (0.970 to 0.979) and 0.974 (0.969 to 0.979). To measure the repeatability across the different plates, the inter-assay ICC was calculated based on the five IRCs, resulting in an ICC of 0.968 (0.909 to 0.986). Telomere length was adjusted for plate effects.

### 2.4 Temperament in infants and preschoolers

Temperament of the infants was reported by the mother with the very short form of the revised Infant Behavior Questionnaire (IBQ-R-vsf) (59, 60) at 3–5 months of age, in Dutch. The IBQ-R-vsf measures infant temperament using 37 items about the frequency of certain behaviors in specific situations (playing, bathing, etc.) of the previous week (e.g., ‘When put into the bath water, how often did the baby smile?’). Items are scored on a seven-point Likert scale (1 = never, 7 = always), containing the factors surgency, negative affectivity and orientation/regulation. See Supplementary eMethods 3 for more information. In our sample the IBQ-R-vsf subscales showed a good internal consistency on all scales (Cronbach’s *α* = between 0.70 and 0.90).

In preschoolers (4 year-olds), a Dutch version (61, 62) of the very short form of the Children’s Behavior Questionnaire (CBQ-R-vsf) from Putnam and Rothbart was used (63, 64), which has scales similar to the IBQ-R-vsf (65), but adjusted for the older age group. See Supplementary eMethods 4 for details. Participation rate of the follow up was 77.6%.

### 2.5 Statistical analysis

All statistical analyses were performed using R version 4.4.2 (R Core Team, Vienna, Austria), and the cutoff value for significance was set at a *p* value < 0.05.

Using multiple linear regression model analyses, we examined the determinants of infant telomere length, with maternal psychosocial and work-related factors during pregnancy as predictors. Finally, we examined infant buccal telomere length as a predictor of infant and preschooler temperament. In order to avoid bias due to missing data (Supplementary eTable 2), we performed multiple imputation using a predictive mean matching procedure using the *mice* R package (66). Imputation was done on the variables relating to the psychosocial and work-related factors, with the latter being excluded from participants who did not work. In total, 105 datasets were generated with a maximum of 20 iterations. Afterwards, imputed datasets were merged using the *merge_imputations* function from the *sjmisc* R package (67), resulting in the dataset for further analyses. As the follow-up date was not present for all mother-infant pairs (*n* = 39 missing), a proximal follow-up date was calculated based on the follow-up date of the previous mother-infant pair, as several pairs usually participated the same day. The models were adjusted for maternal education level, during infancy, age and offspring sex and age at the time of follow up. Education level was defined as a high school or vocational degree and a college or university degree. The effect of sex was examined by interaction terms and stratification of analyses based on sex. Since social satisfaction may also be influenced by the working environment, a final analysis examined the influence of social satisfaction adjusted for QEEW physical demands, COPSOQ emotional demands and COPSOQ developmental opportunities.

**Figure 2:**
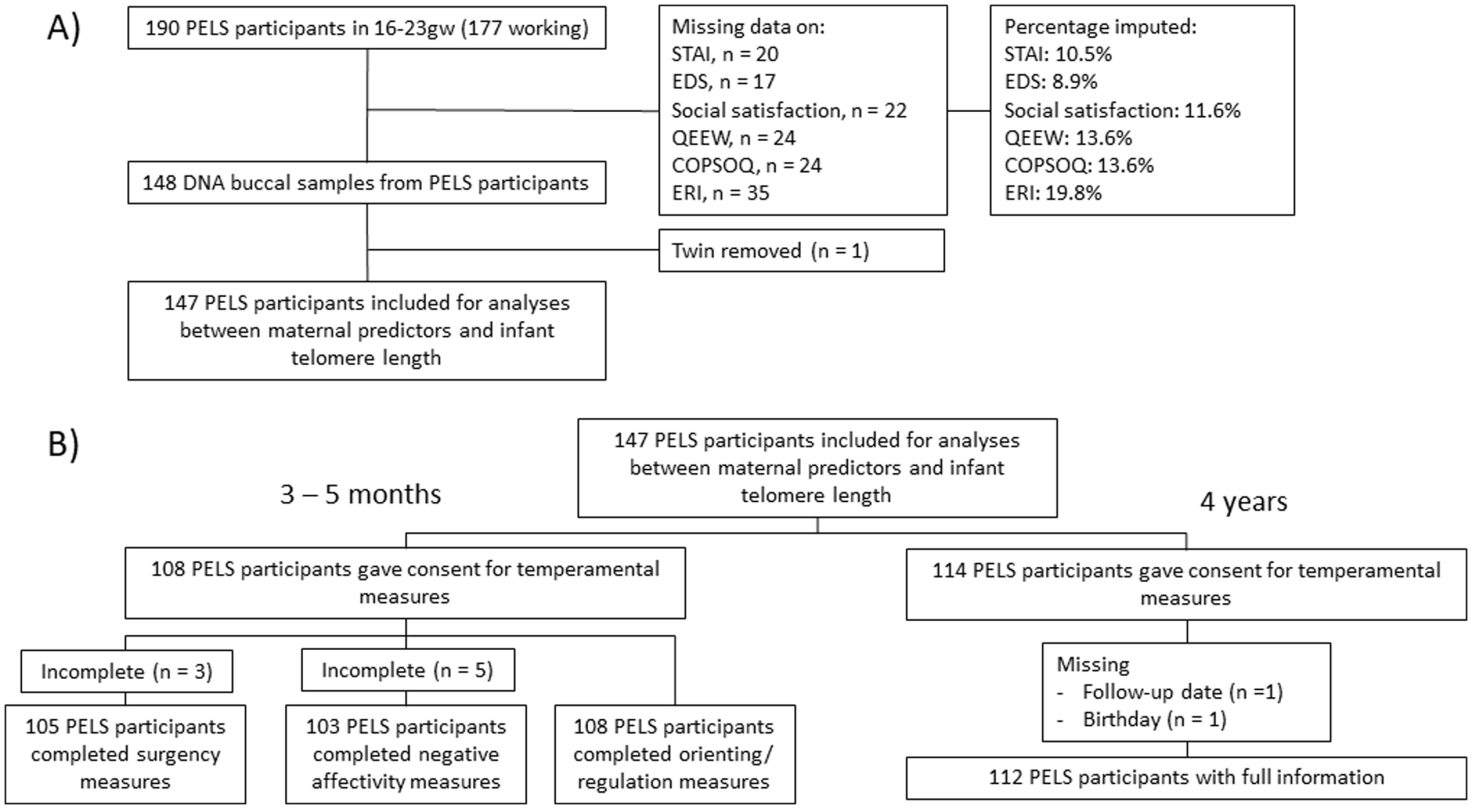
Flowchart of the number of participants in the PELS study cohort used for analyses of **(A)** maternal stressors as predictors of infant telomere length and **(B)** telomere length as predictor of offspring temperament. ‘Gw’, gestational week; STAI, Spielberger State Trait Anxiety Inventory; EDS, Edinburgh Depression Scale; QEEW: Experience and Evaluation of Work; COPSOQ, Copenhagen Psychosocial Questionnaire; ERI-Q, Effort-Reward Imbalance Questionnaire.

Samples were removed due to missing data, genetically relatedness or violation of normality assumption (Figure 2), ending with *n* = 147. A power analysis for the general linear model, using the R package *pwr* (68), showed an effect size of 0.091 would be possible with 80% power, *n* = 147 and *α* = 0.05 for analyses between maternal predictors and infant telomere length, while the effect size would be 0.132 and 0.121 for analyses with infant and preschooler temperament as outcome.

## 3 Results

The descriptive statistics of the study population are summarized in Table 1. Out of the 190 mothers that participated during pregnancy, 147 mother-infant dyads with telomere length data participated at 3–5 months of age. The mothers had a mean (SD) age of 32.1 years (±3.9). Most mothers (*n* = 104, 70.7%) obtained a college or university degree, and worked within the healthcare or education sector (32.0%) (Supplementary eTable 3). Most participating mothers were married or living with their partner (*n* = 137, 93.2%), while n = 2 (1.4%) were single. The mean (SD) maternal BMI was 24.3 (±4.1). The study population contained relatively few preterm births (gestational age less than 36 weeks, *n* = 4) and infants with low birth weights (birth weights < 2,500 g, *n* = 5). Almost all participants were Caucasian, with 3.2% (*n* = 6) being non-Caucasian.

**Table 1:** An overview of the study characteristics in the PELS birth cohort.

Characteristic	Frequency/Mean	%/SD	*n*
Maternal			
Maternal age, years	32.1	3.9	147
BMI, kg/m^2^	24.3	4.1	147
Maternal education			
High school or vocational	43	29.3	147
College or university	104	70.7	147
Marital status			
Married	79	53.7	147
Living-together-contract	29	19.7	147
Living with partner	29	19.7	147
Registered partner	8	5.4	147
Single	2	1.4	147
Working status per 1st trimester			
Working	139	94.6	147
Not working	8	5.4	147
Infant			
Birthweight, grams	3,425	486.0	143
Gestational age, weeks	39.6	1.5	140
Sex			
Boys	71	48.3	147
Girls	76	51.7	147
Age at baseline and follow up			
Age, months, median (IQR)	4	3.5	147
Age, years	4.0	0.2	112

**Figure 3:**
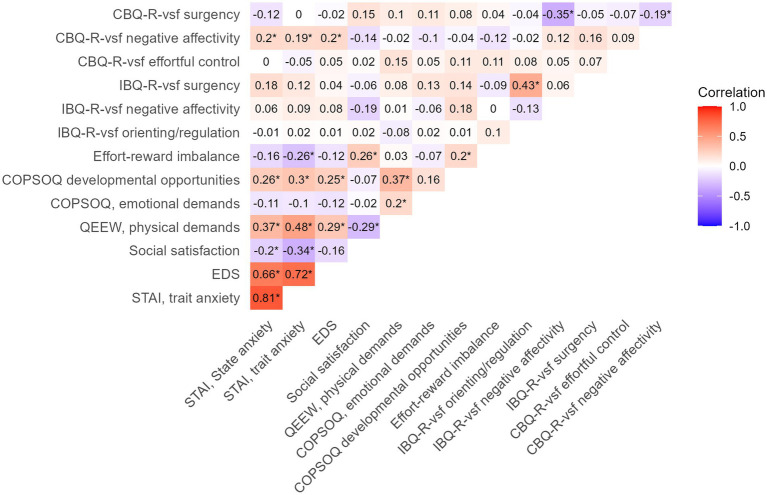
Pearsons’s correlation plot of maternal predictors (STAI, EDS, social satisfaction, effort-reward imbalances, QEEW, COPSOQ) and offspring temperament (IBQ-R-vsf and CBQ-R-vsf). * = Pearson correlation *p* value ≤ 0.05. STAI, State–Trait Anxiety Inventory; EDS, Edinburgh Depression Scale; QEEW, Questionnaire on the Experience and Evaluation of Work; COPSOQ, Copenhagen Psychosocial Questionnaire; IBQ-R, Infant Behavior Questionnaire Revised very short form; CBQ-R, Child Behavior Questionnaire Revised very short form.

The descriptive statistics for all maternal psychosocial and work-related factors and offspring temperament are summarized in Table 2. The mean (SD) maternal state anxiety score during pregnancy was 31.8 (±7.8), and the trait anxiety score was 32.9 (±7.0), with n = 14 participants (9.5%) having a state anxiety score above the threshold for severe anxiety symptoms (state anxiety score ≥ 43). The mean (SD) Edinburgh Depression Scale (EDS) score during pregnancy was 4.1 (±3.0), with 8 (5.4%) participants having EDS scores above the threshold for severe depressive symptoms (EDS score ≥ 12). The mean (SD) satisfaction with social life score during pregnancy was 8.0 (±1.1). Some of the predictors (maternal psychosocial and work-related factors) and temperamental outcomes were correlated (Figure 3). More specifically, maternal EDS and both STAI state and trait anxiety positively correlated with preschooler negative affectivity (Pearson’s *r* = 0.20, 0.20, and 0.19, respectively, all *p*’s ≤ 0.05).

**Table 2:** Descriptive characteristics of maternal variables in pregnancy and offspring temperament.

Factor	Scale	Variable	Time-point	*n*	Mean	Median	SD	1st Q	3rd Q	IQR	Min theoretical. Value	Max theoretical value
Psychosocial (maternal)	STAI	State Anxiety	16-23gw	147	31.8	31.0	7.8	27.0	35.0	8.0	20	80
		Trait Anxiety	16-23gw	147	32.9	32.0	7.0	27.5	37.0	9.5	20	80
	EDS	EDS score	16-23gw	147	4.1	3.0	3.7	1.0	6.0	5.0	0	30
	SOCIAL	Social satisfaction	16-23gw	147	8.0	8.0	1.1	7.0	9.0	2.0	3	9
Work (maternal)	QEEW	Physical demands	16-23gw	139	13.0	11.0	6.2	8.0	16.0	8.0	0	100
	COPSOQ	Emotional demands	16-23gw	139	34.2	33.3	24.8	16.7	50.0	33.3	0	100
		Development	16-23gw	139	77.5	81.2	17.8	68.8	93.8	25.0	0	100
	ERI	ERI score	9-15gw	138	0.5	0.5	0.2	0.4	0.6	0.2	0.25	4
Temperament (offspring)	IBQ-R-vsf	Orienting/regulation	3-5mo	108	5.5	5.6	0.6	5.2	5.9	0.7	1	7
		Negative affectivity	3-5mo	103	2.9	2.7	0.9	2.2	3.4	1.2	1	7
		Surgency	3-5mo	105	3.8	3.9	0.9	3.2	4.5	1.3	1	7
	CBQ-R-vsf	Effort-control	4y	112	4.8	4.9	0.7	4.3	5.3	1	1	7
		Negative affectivity	4y	112	3.1	3.1	0.8	2.5	3.6	1.1	1	7
		Surgency	4y	112	4.0	4.0	0.7	3.6	4.4	0.8	1	7

STAI, State–Trait Anxiety Inventory. EDS, Edinburgh Depression Scale; SOCIAL, the social satisfaction questionnaire; QEEW, Questionnaire on the Experience and Evaluation of Work; COPSOQ, Copenhagen Psychosocial Questionnaire; ERI, Effort-Reward Imbalance; IBQ-R-vsf, Infant Behavior Questionnaire Revised Very Short Form; CBQ-R-vsf, Child Behavior Questionnaire Very Short Form; gw, gestational weeks; y, years; Q, quartile.

The majority of mothers (*n* = 139, 94.6%) were working during gestational week 16–23. As for the work-related stressors during pregnancy, the mean (SD) maternal QEEW physical demands was 13.0 (±6.2), while the COPSOQ score for the emotional demands’ subscale was 34.2 (±24.8), and for the developmental opportunities subscale, the mean (SD) score was 77.5 (±17.8). The mean ERI score was 0.5 (0.2).

The mean (SD) scores of infant surgency, negative affectivity and orienting/regulation (IBQ-R-vsf) were 3.8 (±0.9), 2.9 (±0.9) and 5.5 (±0.6), respectively. At preschooler age, 112 mother-offspring pairs participated (Figure 3). The mean (SD) preschooler surgency, negative affectivity and effortful control scores (CBQ-R-vsf) were 4.0 (±0.7), 3.1 (±0.8) and 4.8 (±0.7), respectively.

The participants during infancy had slightly lower QEEW physical demands and ERI scores during pregnancy, as compared to the non-participants (Supplementary eTable 4). As for participation during preschooler years, the participants had slightly higher STAI state and trait scores during pregnancy, as compared to the non-participants at this stage (Supplementary eTable 4).

Maternal psychosocial and work-related factors were not found to be significantly associated with infant buccal telomere length (Table 3) (*p* values ≥ 0.11).

**Table 3:** Predictors of infant buccal telomere length.

Scales	Estimate (%)	95% CI	*p*	*n*
STAI				
State anxiety	−0.11	−0.69 to 0.48	0.724	147
Trait anxiety	−0.31	−0.95 to 0.33	0.342	147
Edinburgh depression scale	−0.42	−1.63 to 0.80	0.498	147
Social satisfaction	3.24	−0.75 to 7.22	0.111	147
QEEW physical demands	−0.34	−1.18 to 0.49	0.414	139
COPSOQ				
Emotional demands	−0.04	−0.23 to 0.16	0.712	139
Developmental opportunities	0.02	−0.26 to 0.29	0.917	139
Effort-reward imbalance	−18.05	−41.26 to 5.16	0.126	138

Estimates from several linear model analyses are represented as estimate per unit increase in the predictor. The models were adjusted for plate effects, maternal age, education level and BMI and infant sex and age at the time of sampling. STAI, State–Trait Anxiety Inventory; EDS, Edinburgh Depression Scale; QEEW, Questionnaire on the Experience and Evaluation of Work; COPSOQ, Copenhagen Psychosocial Questionnaire; CI, Confidence Interval.

We did not observe any significant associations between infant buccal telomere length and temperamental measures at 3–5 months or 4 years of age (Table 4). Stratifying the analyses by sex did not show any associations (all *p*’s ≥ 0.16) (Supplementary eTable 5).

**Table 4:** Infant buccal telomere length as a predictor of offspring temperament.

Outcome	Time-point	Est. (score)	95% CI	*p*	*n*
IBQ-R-vsf					
Orienting/ regulation	3-5mo	0.18	−0.25 to 0.62	0.404	108
Negative affectivity	3-5mo	−0.14	−0.85 to 0.56	0.686	103
Surgency	3-5mo	0.13	−0.45 to 0.71	0.660	105
CBQ-R-vsf					
Effortful control	4y	0.01	−0.48 to 0.51	0.962	112
Negative affectivity	4y	−0.28	−0.86 to 0.30	0.335	112
Surgency	4y	0.38	−0.08 to 0.84	0.107	112

Estimates from several linear regression model analyses are represented as score differences in the temperamental outcome per percent increase in infant telomere length. The models were adjusted for plate effects, maternal age, education level and BMI and offspring sex and age at the time of sampling. Est., Estimate; IBQ-R-vsf, Infant Behavior Questionnaire Revised Very Short Form; CBQ-R-vsf, Child Behavior Questionnaire Very Short Form; CI, Confidence Interval; IQR, Interquartile range; mo, months; y, years.

Further adjusting the social satisfaction model for work-related stressors (QEEW physical demands and COPSOQ emotional demands and COPSOQ developmental opportunities) showed a 4.2% (95% CI: −0.32 to 8.76%, *p* = 0.070) higher buccal telomere length during infancy per score increase in social satisfaction. See Table 5 for the full model. This result was unchanged after removal of imputed data (4.2, 95% CI: −0.32 to 8.76%, *p* = 0.071) in a sensitivity analysis. However, after including the ERI score in the model, the corresponding estimate dropped to 3.40% (95% CI: −1.37 to 8.18, *p* = 0.16).

**Table 5:** Maternal social satisfaction as predictor of infant telomere length after adjustment for work-related stressors.

Variables	Estimate (%)	95% CI	*p*
Social satisfaction	4.22	−0.32 to 8.76	0.070
QEEW physical demands	−0.38	−1.24 to 0.48	0.390
COPSOQ			
Emotional demands	0.002	−0.20 to 0.20	0.981
Developmental opportunities	−0.06	−0.35 to 0.23	0.702
Educational level			
High school or vocational	Ref	Ref	Ref
College or university	2.98	−8.78 to 14.73	0.621
Maternal BMI	−0.55	−1.69 to 0.59	0.345
Maternal age	0.49	−1.8 to 0.82	0.465
Infant sex			
Boy	Ref	Ref	Ref
Girl	0.36	−9.24 to 9.96	0.942
Infant age	−0.02	−0.09 to 0.14	0.679

Variables and estimates from one linear regression model are shown as a percentage change in infant plate-adjusted telomere length per unit increase in the variable. Ref = reference category. STAI, State–Trait Anxiety Inventory; EDS, Edinburgh Depression Scale; QEEW, Questionnaire on the Experience and Evaluation of Work; COPSOQ, Copenhagen Psychosocial Questionnaire; CI, Confidence Interval. *N* = 138.

## 4 Discussion

This study explored the associations between maternal psychosocial and work-related factors during pregnancy and infant buccal telomere length at 3–5 months of age, and with temperament in infancy and preschool years (4 years of age). Overall, we did not observe significant associations between maternal predictors and infant buccal telomere length or between infant buccal telomere length and offspring temperament. However, after adjusting social satisfaction for work-related stressors, we found a trend for social satisfaction to have a beneficial impact on infant telomere length.

The lack of associations between single-predictor maternal predictors and infant buccal telomere length was unexpected, given previous evidence linking maternal stress during pregnancy to infant telomere length (28). For example, associations between STAI state and trait scores with cord tissue telomere length have been reported in studies with roughly 890 and 1,400 mother–child pairs (17, 69), although, for one study, this did not pass FDR correction (69). However, in a large cohort study including 4,299 children at the age of 4–5, no association was found between maternal depression assessed 9 months after birth, measured by the EDS, and child telomere length, even with 15% of participants having elevated depression scores (EDS score ≥ 13) (70). The same applied to another study with 1,400 newborn, using the Center for Epidemiological Studies Depression Scale (CES-D) (69). Similarly, as in our study, other research has not found a consistent link between maternal depression and offspring telomere length (71–73). While one study identified a positive relationship between maternal resilience, incorporating social support, and cord blood telomere length (74), another observed no such association in a smaller sample setting, using salivary telomere length (75). A recent meta-analysis further concluded that social support was not significantly associated with telomere length in adults, underscoring the mixed findings in this area (76). While our findings in infants showed a trend for a positive influence of maternal social satisfaction in pregnancy, this was only observed after accounting for work-related stressors. This result underscores the importance of taking both occupational and residential stressors and buffers into account.

Despite our lack of associations between maternal psychosocial factors and infant telomere length, we found maternal depressive symptoms (EDS) and state and trait anxiety (STAI) during pregnancy to be positively correlated with negative affectivity in preschoolers. This pattern suggests that maternal psychosocial factors may influence offspring temperament through other pathways than telomere length. Indeed, we did not find any associations between buccal telomere length and temperamental measures. Other studies suggest a connection between telomere length in adults and impulsivity (5), hostility (3), and some personality traits (4). In 607 infants, buccal telomere length was found to be associated with regulation/effortful control, and infancy surgency to lessen buccal telomere length attrition until the age of 3 years (34). Compared to our study, this study had higher scores of negative affectivity (2.9 versus 3.2), and surgency (3.8 vs. 4.7), and lower regulation/orientation scores (5.5 versus 4.8). Considering the relatively large difference in surgency scores, higher scores may be needed for a noticeable change in telomere length to occur.

The direction of association between telomere length and behavior is unknown and might be explained by complex interactions. Some studies tend to view behavior as influencing telomere length change (77, 78), stating that the mechanism behind telomere length affecting behavior is unclear (79). Indeed, behavior affecting telomere length is more approachable, for instance, impatient or risky behavior being more likely to, e.g., start smoking or eating unhealthy (5), resulting in increased oxidative stress and hence increased telomere length vulnerability. Considering that the ability to do such is unlikely during infancy, telomere length may influence behavior. One possible mechanism is through the telomere position effect (TPE), the spreading of heterochromatin from the telomeric region into nearby genes, causing their silencing (80). Silencing of genes over longer distances can also happen by the telomeric ends looping back onto the chromosomes, called TPE over long distances (TPE-OLD) (81). As far as we know, no TPE-OLD affected genes have been found to be involved in behavior (82), but the potential for this to happen still stands. A third possibility is the influence of a shared factor on both telomere length and behavior. For instance, an inflammatory process in the body may influence temperament (83–85) and cause increased attrition of telomere length, making telomere length an indirect marker of inflammation or other conditions that cause oxidative stress. At the moment of writing, telomere length influencing behavior is still unclear and needs further research.

This study has several strengths and limitations. First of all, the study examined a rather unknown area of research, particularly the effects of maternal social life satisfaction and work-related stressors during pregnancy and offspring temperament in relation to infant buccal telomere length. The longitudinal aspect of the study, temperamental analysis at 3–5 months and 4 years of age, furthermore aids our understanding of how telomere length in the early life can influence temperament, and possible risk of neurocognitive issues and behavioral problems in the later life. However, we recognize a limitation in variance of both socioeconomic class, with no participating mothers having a low educational attainment, and stress. The mean (SD) STAI state and trait scores have previously been reported as 34.6 (9.8) and 36.7 (8.9), respectively (17), which were slightly higher than in our study of 31.9 (7.8) and 32.9 (7.0). The depression score, as assessed via the EDS, was found to be low in the PELS cohort, with a mean (SD) of 4.1 (3.0) during the 2nd trimester. In comparison, the ALSPAC cohort presented with a mean (SD) of 6.99 (4.87) at gestational week 21 (86). Additionally, the PELS cohort reported slightly more favorable working conditions than previously described, with mean (SD) scores of 34.2 (24.8) and 77.5 (17.8) for emotional demands and developmental opportunities, compared with 37.8 (25.5) and 68.5 (18.4), respectively, from 1603–1850 participants from the establishment of the COPSOQ (50). A greater variation in the stressors examined might have revealed more associations. Additionally, 12–27% of the data included imputed values, which could introduce uncertainties in the analyses. However, studies have shown listwise deletion to introduce an even greater bias (87), with 5% missing data being the rule of thumb for when complete-case analysis no longer suffices. Additionally, since most maternal predictors (psychosocial and work-related factors) and offspring temperament were assessed through maternal self-report, there is a potential for rater bias. Validation from an additional informant, such as the other parent or a professional, or standardized observational assessments of offspring behavior, might have provided more robust measures. However, while the interparent agreement for the IBQ-R-vsf has a mean of 0.41, the retest reliability is high, being 0.72 on average, if only completed by the mother (60).

## 5 Conclusion

Infant buccal telomere length was not associated with prenatal exposure to maternal psychosocial or work-related factors in the single-predictor models, nor with temperament later in life. However, prenatal exposure to maternal social satisfaction may have a beneficial impact on infant telomere length. Further research is needed to explore these associations and take into account both occupational as well as residential stressors and buffers.

## Data Availability

The raw data supporting the conclusions of this article will be made available by the authors, without undue reservation.
