# Supplementary material for: Infant buccal telomere length: associations with maternal distress in pregnancy and offspring temperament
Source: Front Public Health. 2025 Oct 21;13:1657714. doi: 10.3389/fpubh.2025.1657714 (PMC12584428; doi:10.3389/fpubh.2025.1657714)
Supplement: Supplementary file 1 [file Data_Sheet_1.pdf]

Supplementary Material

Contents

Supplementary eMethods 1: Effort-Reward Imbalances. 2

Supplementary eMethods 2: Telomere length measurements 3

Supplementary eMethods 3: Infant Behavior Questionnaire (IBQ-R-vsf) 4

Supplementary eMethods 4: Children's Behavior Questionnaire (CBQ-R-vsf) 5

Supplementary eTable 1: Yield and purity of the DNA samples extracted from buccal swaps from the infants of the PELS cohort 6

Supplementary eTable 2: Overview of number of imputed data 7

Supplementary eTable 3: The job sectors the PELS participants worked in during pregnancy (n = 147) 8

Supplementary eTable 4: PELS study population comparisons at baseline (measurements during pregnancy) and follow-up 9

Supplementary eTable 5: Sensitivity analyses of the influence of sex................................... 10

Supplementary eMethods 1: Effort-Reward Imbalances.

The Effort-Reward Imbalances (ERI) was used during pregnancy. Six areas relating to the effort or burden of work were examined, with yes/no questions such as ‘I often have to work overtime’. If the reply was ‘yes’, a follow-up 1 – 5 scale of the intensity was supplied, going from ‘Little to no effort’ to ‘Very high effort/burden’. The score ranges from 6 – 24. Reward was measured in 10 areas, relating to appreciation, payment and job security, with questions such as ‘I get the appreciation I deserve from my colleagues’. If the reply was ‘no’ to a positive question, as in the example, or ‘yes’ to negative question, the participant was asked to use the 1 – 5 scale to indicate the extend. The reward score ranges from 10 to 40. The Effort-Reward ratio (ERI) was calculated by effort/(reward x correction factor), where the correction factor was used to correct for the different number of items in the effort and reward-part of the questionnaire. A ratio of 1 would equal one effort per reward, a ratio higher than 1 would mean a higher reward for the same effort, and vice versa, a ratio lower than 1 would signify more effort per reward.

Supplementary eMethods 2: Telomere length measurements

The CNRQ is calculated firstly by using the delta-Cq method to calculate the relative quantity (RQ) of telomere and HBG genes, using the arithmetic mean of all Cq values of their respective genes in order to minimize measurement error. The RQs are then divided with each other, yielding the normalized relative quantities. To account to run-to-run variation, the calibration factor is calculated by taking the geometric mean of the normalized relative quantities of the IRCs per plate, whereafter the normalized relative quantities of the samples are divided by their plate-specific calibration factor, yielding the final T/S ratios (CNRQs). The amplification efficiency was on average (SE) 97.4% (0.48%) for the telomere plates and 91.5% (1.11%) for the HBG plates. The mean R^2 was 0.998, with y-intercepts from CT 16.006 to 16.828 for the telomere plates, and from CT 24.982 to 26.104 for the HBG plates. The mean Cq of the telomere NTC's was 23.396. The HBG NTC's did not amplify.

Supplementary eMethods 3: Infant Behavior Questionnaire (IBQ-R-vsf)

The IBQ-R-vsf was used at the age of 3 – 5 months. A second order factor analysis on the subscales scores of the original IBQ-R (long version) resulted in the following three broad dimensions that are also used for the short version of the IBQ-R: surgency (13 items), negative affectivity (12 items), and orientation/regulation (13 items). Surgency/extraversion is defined as a combination of low shyness and high approach (e.g., 'During a peek-a-boo game, how often did the baby smile?') and has been linked to the personality characteristics 'agreeableness' and 'extraversion'. Negative affectivity is defined as the tendency for an offspring to experience negative feelings (e.g., 'When placed on his/her back, how often did the baby fuss or protest?') and has been associated with neuroticism and negative emotionality in adults. Finally, orienting/regulation is defined as the tendency of an infant to regulate itself, and is linked with low intensity pleasure (e.g., 'When playing quietly with one of his/her favorite toys, how often did the baby show pleasure?') and duration of orienting (e.g., 'How often during the last week did the baby stare at a mobile, crib bumper or picture for 5 min or longer?') and has been linked to the personality characteristic 'conscientiousness' in adults.

Supplementary eMethods 4: Children's Behavior Questionnaire (CBQ-R-vsf)

The CBQ-R-vsf was used to asses behavior at the age of 4 years. The scale differences between the IBQ-R and CBQ-R are content of items and name of the scales that suit the older age group ("duration of orienting" to "attention focusing" and "rate of approach" to "positive anticipation"), removal of scales in the CBQ-R ("affiliation/cuddliness") and addition of scales in the CBQ-R ("discomfort", "impulsivity", "inhibitory control" and "shyness"). The CBQ-R-vsf contains 36 items with statements about the child, where the mother rates the fit on seven-point Likert scale, going from 1 (extremely untrue) to 7 (extremely true). As with the IBQ-R-vsf, the CBQ-R-vsf contain three broad dimensions, being: surgency/extraversion, negative affectivity and effortful control, with the latter being the same as regulating/orienting in the IBQ-R, but with the added capability for the child to shift focus and plan ahead. Examples for each dimension include "my child likes going down high slides or other adventurous activities", "my child gets angry when s(he) can't find something s(he) wants to play with" and "my child approaches places (s)he had been told are dangerous slowly and cautiously."

Supplementary eTable 1: Yield and purity of the DNA samples extracted from buccal swaps from the infants of the PELS cohort

Function	ng/uL	A260/A280	A260/A230
Mean	45.035	1.879	1.640
Median	31.835	1.900	1.630
Standard deviation	43.007	0.162	0.385
Interquartile range	25.505	0.225	0.515
Quartile 1	22.895	1.765	1.410
Quartile 3	48.400	1.990	1.925

Supplementary eTable 2: Overview of number of imputed data

Factor	Scale	Variable	Timepoint	Imputed n	Percentage imputed
**Psychosocial (maternal) (n = 190)**	**STAI**	State anxiety	16-23gw	20	10.5
		Trait anxiety	16-23gw	20	10.5
	**EDS**	EDS score	16-23gw	17	8.9
	**Social**	Social satisfaction	16-23gw	22	11.6
**Work (maternal) (n = 177)**	**QEEW**	Physical demands	16-23gw	24	13.6
	**COPSOQ**	Emotional demands	16-23gw	24	13.6
		Development	16-23gw	24	13.6
	**ERI**	ERI score	9-15gw	35	19.8

STAI = State-Trait Anxiety Inventory. EDS = Edinburgh Postnatal Depression Scale, Social = the social satisfaction questionnaire, QEEW = Questionnaire on the Experience and Evaluation of Work, COPSOQ = Copenhagen Psychosocial Questionnaire, ERI = Effort-Reward Imbalance, IBQ-R-vsf = Infant Behavior Questionnaire Revised Very Short Form, gw = gestational weeks

Supplementary eTable 3: The job sectors the PELS participants worked in during pregnancy (n = 147)

Job sector	Number	Percent
Healthcare and welfare	47	31.97
Education	24	16.33
Financial	13	8.84
Other	13	8.84
Business and IT	12	8.16
Not working	8	6.12
Public administration	8	5.44
Retail	6	4.08
Construction	4	2.72
Industry and manufacturing	4	2.72
Transport and telecommunications	2	1.36
Unknown	1	0.68
Wholesale	1	0.68
Hospitality/catering	1	0.68
Agriculture and fisheries	1	0.68
Utilities	1	0.68
Tourism	1	0.68

Supplementary eTable 4: PELS study population comparisons at baseline (measurements during pregnancy) and follow-up

Variable	Baseline characteristics versus participation during infancy follow-up			Baseline characteristics versus participation during preschooler follow-up		
	Participation	No participation		Participation	No participation	
	Median (10th-90th percentile)	Median (10th-90th percentile)	P	Median (10th-90th percentile)	Median (10th-90th percentile)	P
Maternal age, y	32.0 (28.0 to 37.6)	32.5 (26.1 to 35.9)	0.284	32.0 (28.0 to 38.0)	34.0 (29.0 to 36.0)	0.650
BMI, kg/m^2	23.4 (20.4 to 28.0)	23.5 (20.0 to 29.9)	0.870	23.5 (20.2 to 28.3)	23.4 (20.5 to 27.4)	0.959
Maternal education			0.347			1
High school	31 (26.96%)	12 (37.50%)		33 (28.95%)	10 (30.30%)	
College/university	84 (73.04%)	20 (62.50%)		81 (71.05%)	23 (69.70%)	
STAI, state anxiety	31.0 (23.0 to 40.8)	31.0 (21.1 to 39.9)	0.740	30.5 (22.0 to 38.7)	33.0 (28.0 to 48.0)	0.037
STAI, trait anxiety	32.0 (25.0 to 42.0)	30.0 (25.0 to 46.5)	0.563	31.0 (25.0 to 40.4)	35.0 (27.2 to 46.8)	0.016
Edinburgh depression scale	4.0 (1.0 to 8.0)	2.5 (0.0 to 8.0)	0.103	3.0 (0.0 to 8.0)	4.0 (1.0 to 13.6)	0.152
Social satisfaction	8.0 (6.0 to 9.0)	8.0 (6.0 to 9.0)	0.230	8.0 (6.0 to 9.0)	8.0 (6.0 to 9.0)	0.883
Effort-reward imbalances	0.45 (0.3 to 0.7)	0.53 (0.4 to 0.7)	0.058	0.45 (0.3 to 0.7)	0.51 (0.4 to 0.8)	0.052
QEEW, physical demands	11.0 (7.0 to 21.0)	15.0 (7.0 to 26.2)	0.038	11.0 (7.0 to 21.6)	11.0 (7.0 to 21.0)	0.490
COPSOQ, emotional demands	33.33 (0 to 75)	33.33 (0 to 66.67)	0.811	33.33 (0 to 75)	33.33 (0 to 66.67)	0.591
COPSOQ developmental opportunities	81.25 (50 to 93.75)	75 (55.62 to 94.38)	0.431	81.25 (50 to 93.75)	81.25 (50 to 93.75)	0.304

Significance tests were conducted based on whether the variable was normally distributed (two-sided t-test), non-normally distributed (Wilcoxon test), as determined by a Shapiro Wilk test, or categorical (Chi-square test). QEEW = Questionnaire on the Experience and Evaluation of Work, COPSOQ = Copenhagen Psychosocial Questionnaire

Supplementary eTable 5: Sensitivity analyses of the influence of sex

		Boys				Girls				Interaction	
**Predictors**	**Outcomes**	**β (%)**	**95% CI**	**p**	**n**	**β (%)**	**95% CI**	**p**	**n**	**p**	**n**
**STAI**											
**State anxiety**	**TL**	-0.123	-0.90 to 0.66	0.753	71	-0.17	-1.13 to 0.80	0.727	76	0.761	147
**Trait anxiety**	**TL**	-0.25	-1.10 to 0.61	0.568	71	-0.44	-1.46 to 0.58	0.391	76	0.657	147
**EDS**	**TL**	0.16	-1.42 to 1.73	0.845	71	-1.4	-3.46 to 0.66	0.179	76	0.163	147
**Social satisfaction**	**TL**	4.1	-2.18 to 10.37	0.197	71	2.31	-3.26 to 7.87	0.411	76	0.581	147
**QEEW physical demands**	**TL**	-0.37	-1.86 to 1.11	0.616	67	-0.39	-1.46 to 0.68	0.466	72	0.777	139
**COPSOQ**											
**Emotional demands**	**TL**	-0.094	-0.374 to 0.187	0.506	67	-0.018	-0.3 to 0.265	0.900	72	0.787	139
**Developmental opportunities**	**TL**	0.060	-0.334 to 0.454	0.761	67	-0.056	-0.465 to 0.353	0.786	72	0.621	139
**ERI**	**TL**	-14.98	-50.32 to 20.36	0.400	66	-20.3	-52.65 to 12.04	0.215	72	0.684	138
	**IBQ-R-vsf**										
**TL**	**Orienting/ regulating**	0.13	-0.55 to 0.80	0.712	50	0.19	-0.41 to 0.79	0.531	58	0.950	108
**TL**	**Negative affectivity**	0.43	-0.85 to 1.72	0.500	47	-0.45	-1.28 to 0.39	0.288	56	0.237	103
**TL**	**Surgency**	-0.02	-0.99 to 0.97	0.974	50	0.25	-0.50 to 0.99	0.509	55	0.716	105
	**CBQ-R-vsf**										
**TL**	**Effortful control**	0.25	-0.51 to 1.02	0.506	54	-0.20	-0.88 to 0.47	0.547	58	0.435	112
**TL**	**Negative affectivity**	0.11	-0.69 to 0.92	0.778	54	-0.63	-1.49 to 0.22	0.143	58	0.253	112
**TL**	**Surgency**	0.28	-0.36 to 0.91	0.386	54	0.46	-0.24 to 1.15	0.194	58	0.766	112

Several linear regression models, with estimates being expressed as a percentage change in infant buccal telomere length per unit increase in the maternal predictors, and as a score difference in offspring temperament per percent increase in infant buccal telomere length. All models were adjusted for storage time, plate effects, maternal education, BMI and maternal and offspring age at the time of follow up. TL = telomere length, STAI = State-Trait Anxiety Inventory, EDS = Edinburgh Postnatal Depression Scale, QEEW = Questionnaire on the Experience and Evaluation of Work, COPSOQ = Copenhagen Psychosocial Questionnaire, ERI = Effort-Reward Imbalance, IBQ-R-vsf = Infant Behavior Questionnaire Revised Very Short Form, CBQ-R-vsf = Child Behavior Questionnaire Revised Very Short Form
